# Financial Implications of Boarding: A Call for Research

**DOI:** 10.5811/westjem.2021.1.49527

**Published:** 2021-04-09

**Authors:** Maureen M. Canellas, Kevin A. Kotkowski, Sean S. Michael, Martin A. Reznek

**Affiliations:** * University of Massachusetts Medical School, Department of Emergency Medicine, Worcester, Massachusetts; † University of Colorado School of Medicine, Department of Emergency Medicine, Aurora, Colorado

Boarding, the practice of holding patients in emergency departments (ED) after a decision has been made to admit them to the hospital,^1^ is well known to adversely affect patient care. Multiple investigations have shown that boarding negatively impacts quality and patient safety outcomes including mortality,^2^–^7^ readmission rate,^8^ hospital length of stay,^2^,^5^,^8^,^9^ and patient satisfaction.^10^–^12^ In addition, boarding is known to be a major contributor to overall ED crowding,^13^ which also has been demonstrated to have significant negative impact on quality and safety.^13^,^14^ Multiple operational tactics are known to reduce boarding but, concerningly, adoption of them has been inconsistent.^13^,^15^ Also concerning, ED boarding appears to be worsening over time, based upon our unpublished year-over-year review of two large national ED operations benchmarking databases, the Emergency Department Benchmarking Alliance and the Academy of Administrators in Academic Emergency Medicine/Association of Academic Chairs of Emergency Medicine.^16^,^17^

The constellation of boarding having been known to adversely affect patient care outcomes for over two decades, inconsistent implementation of tactics known to reduce boarding, and evidence that boarding may be worsening over time naturally raises questions of the barriers to improvement. Chief among these questions is why implementation of boarding-reduction tactics has not consistently occurred, despite their clear benefits. In that regard, some experts have postulated that financial drivers may be at play.^18^,^19^

To investigate the potential for financial drivers contributing to boarding, we performed a systematic review, pre-registered with PROSPERO (#CRD42016037794). We reviewed 1185 manuscripts from the past five years, and while we identified a number of articles that considered downstream financial implications of ED crowding, only two investigations studied the financial drivers specific to boarding. In 2015, Dyas et al created a cost model formula to estimate the opportunity costs of boarders and the revenue gained from process improvement changes. Using this model, they estimated a $4,000,000 financial benefit by reducing their average boarding time to below 60 minutes. However, the model was highly simplified and only considered the costs associated with a single diagnosis per patient, regardless of their overall condition and other diagnoses.^20^ In 2017, Schreyer and Martin showed that maintaining an admitted patient in an ED bed cost the hospital twice as much as an inpatient bed and five times as much as an admissions holding-unit bed. Notably, this single-center publication assumed that the admissions holding unit could be added to their center’s ED without renovation or building costs.^21^

Drawing valid, generalizable conclusions from just these two studies is not possible; however, we believe that the paucity of research in this area is—in and of itself—meaningful. The bottom line is that there is very little scientific inquiry into the financial effects of boarding; thus, there remain three mutually exclusive possibilities, in our view:

1. Boarding is financially advantageous for hospitals This has been postulated by some experts, citing the fact that fee-for-service Medicare reimbursements are on average $700 more per elective admission than emergent admissions.^22^ If this scenario is demonstrably true, it may prompt consideration of reimbursement reform to incentivize boarding reduction.
2. Boarding is financially disadvantageous for hospitals While it may appear that there are revenue incentives for hospitals to continue to board patients admitted from EDs based on the $700 difference cited above, we hypothesize that there are likely significant and disproportionate cost disadvantages to boarding, potentially masked by limitations in historical cost accounting practices and investigational methodologies.^23^ Recalculating total costs of boarding care would require modifying these structural accounting factors. However, uncovering that boarding is financially disadvantageous would add additional incentive beyond the known quality and safety benefits for hospitals to implement boarding reduction tactics.
3. Boarding is financially neutral for hospitals. It is possible that there are financial advantages and disadvantages of boarding that balance each other. If a financially neutral scenario is demonstrated to be true, the quality and safety evidence alone should then prompt broader adoption of boarding-reduction tactics.

Determining which of the above scenarios holds true will be critical if our society is to address the growing issue of boarding in a socially and financially responsible manner. Historically, financial investigations in healthcare have been challenging due to traditional healthcare cost accounting methods being highly complex, poorly designed,^24^ and fraught with outdated assumptions about the details of care delivery models. We suspect that this phenomenon is likely the root cause of the paucity of research uncovered by our investigation. Nonetheless, promising alternative costing methodologies exist that may overcome these barriers. For example, some have proposed an alternative approach to traditional cost accounting methods known as time-driven activity-based costing (TDABC).^25^ This structured methodology, used rarely in healthcare settings but often in other industries,^26^ could be employed to calculate resources consumed by a patient as they progress through care. This technique holds tremendous potential in boarding-related financial investigations. Conducting a randomized trial of boarding has obvious ethical constraints, but leveraging the fact that boarding naturally varies across patients over time and within any given institution (even within common diagnoses) may allow for prospective investigations using TDABC methodology. Once actual costs are known, computer simulations could enable further research in this important area.

Although accurately determining costs of care is likely the most important component to understanding the financial implications of boarding, there are some additional financial factors that also must be considered. Primarily, boarding patients creates opportunity costs, including ED patients leaving before being seen and reductions in accepted ED transfers and ambulance diversion (where still allowed). This lost revenue also likely extends beyond the ED, as a portion of these patients would require hospital admission and undergo surgical procedures. Likewise, there may be opportunity costs to certain boarding mitigation strategies, such as reducing elective surgeries and elective medical admissions. Negative quality outcomes and excess mortality also have financial costs, and boarding-attributable losses in this realm are often overlooked. The most effective simulation models must account for these additional cost dimensions to fully understand the financial impact of ED boarding.

In conclusion, our recently conducted systematic review demonstrated a paucity of published investigations of the financial impacts of boarding. We believe that this research void is a significant contributing factor in the persistence, and possibly worsening, of the practice of boarding in EDs. Therefore, we suggest an increased focus in this area of research, using methodologically sound techniques such as time-driven activity-based costing and computer simulation.
